# Cross-Complementation Study of the Flagellar Type III Export Apparatus Membrane Protein FlhB

**DOI:** 10.1371/journal.pone.0044030

**Published:** 2012-08-29

**Authors:** Clive S. Barker, Fadel A. Samatey

**Affiliations:** Trans-membrane Trafficking Unit, Okinawa Institute of Science and Technology, Onna, Kunigami, Okinawa, Japan; Louisiana State University and A & M College, United States of America

## Abstract

The bacterial type III export apparatus is found in the flagellum and in the needle complex of some pathogenic Gram-negative bacteria. In the needle complex its function is to secrete effector proteins for infection into Eukaryotic cells. In the bacterial flagellum it exports specific proteins for the building of the flagellum during its assembly. The export apparatus is composed of about five membrane proteins and three soluble proteins. The mechanism of the export apparatus is not fully understood. The five membrane proteins are well conserved and essential. Here a cross-complementation assay was performed: substituting in the flagellar system of *Salmonella* one of these membrane proteins, FlhB, by the FlhB ortholog from *Aquifex aeolicus* (an evolutionary distant hyperthermophilic bacteria) or a chimeric protein (AquSalFlhB) made by the combination of the trans-membrane domain of *A. aeolicus* FlhB with the cytoplasmic domain of *Salmonella* FlhB dramatically reduced numbers of flagella and motility. From cells expressing the chimeric AquSalFlhB protein, suppressor mutants with enhanced motility were isolated and the mutations were identified using whole genome sequencing. Gain-of-function mutations were found in the gene encoding FlhA, another membrane protein of the type III export apparatus. Also, mutations were identified in genes encoding 4-hydroxybenzoate octaprenyltransferase, ubiquinone/menaquinone biosynthesis methyltransferase, and 4-hydroxy-3-methylbut-2-en-1-yl diphosphate synthase, which are required for ubiquinone biosynthesis. The mutations were shown by reversed-phase high performance liquid chromatography to reduce the quinone pool of the cytoplasmic membrane. Ubiquinone biosynthesis could be restored for the strain bearing a mutated gene for 4-hydroxybenzoate octaprenyltransferase by the addition of excess exogenous 4-hydroxybenzoate. Restoring the level of ubiquinone reduced flagella biogenesis with the AquSalFlhB chimera demonstrating that the respiratory chain quinone pool is responsible for this phenomenon.

## Introduction

The bacterial flagellum is an intricate nanomachine, which is made of about 30 different proteins [1]. It rotates like a propeller to enable the cells to swim [2], [3]. At the base of the flagellum a type III export apparatus is proposed to exist for the secretion of the external protein components. The type III secretion system, sometimes referred to as the needle complex, which is found in some pathogenic Gram-negative bacteria also uses a type III export apparatus. This highly homologous export apparatus is used to inject proteins for infection into Eukaryotic host cells, which is a major source of pathogenicity [4], [5]. The proton motive force is the energy source for the export apparatus of both flagellar assembly and the needle complex, and in bacteria such as *Escherichia coli* and *Salmonella enterica* serovar Typhimurium (*S. typhimurium*) it is the energy source for the rotation of the fully-formed flagellum [1], [6], [7].

The bacterial flagellar export apparatus consists of about five membrane proteins and three soluble proteins. The membrane proteins are highly conserved with a high sequence identity from bacterial species to species, and to the export apparatus of the needle complex [8]. These proteins are called FlhA, FlhB, FliP, FliQ and FliR. They are essential for protein export. In the *Salmonella* flagellar export apparatus there is a less conserved and non-essential sixth membrane protein, FliO [9]. It has no homolog in the virulence-associated needle complexes and is absent from flagellar systems of some bacteria [3]. The *Salmonella* flagellar export system also contains three soluble proteins, FliH, FliI, and FliJ. FliI is an ATPase, which shows structural similarity to the α and β subunits of F_0_F_1_-ATP synthase [10]. FliI is proposed to use the energy from ATP hydrolysis to enable flagellar protein release into the pore of the secretion apparatus.

FlhA (75 kDa) and FlhB (42 kDa) have membrane-spanning N-terminal regions, which are predicted to have 8 trans-membrane α-helices for FlhA and 4 trans-membrane α-helices for FlhB [1]. Both FlhA and FlhB have large C-terminal cytoplasmic domains, which act as docking platforms for exported protein substrates and for soluble protein components of the export apparatus [11]–[13]. Delivery of FliJ to FlhA by the FliH-FliI complex converts the export apparatus into an efficient membrane potential-driven protein exporter [14]. FlhB coordinates the switch in export from rod/hook to filament type protein substrates through autocleavage within its cytoplasmic domain at a highly conserved ‘Asn-Pro-Thr-His’ cleavage site, between the asparagine and proline residues [15]. The timing of the switch from rod/hook to filament type protein substrates also involves the soluble protein FliK, which measures the length of the hook and when the hook reaches approximately 55 nm a switch is made to filament type proteins [16], [17].

Although the proteins of the flagellar export apparatus have been well studied it is still not fully understood how they function together as a protein exporter. In particular, little is presently known regarding the function of the membrane-embedded regions of the proteins of the type III export apparatus. This is in part because of the difficulty with studying membrane proteins biochemically, and in particular due to their poor stability and the necessity of using detergents to maintain solubility. Genetic analysis of membrane proteins of the flagellum or needle complex has yielded interesting results, however, for example analysis of extragenic suppression has been used to study function of the membrane-embedded MotA and MotB proteins of the bacterial flagellar motor [18]–[20]. Also, suppressor mutations encoded within FlhA were able to rescue motility of strains bearing mutations within FliF, which comprises the MS ring that is presumed to surround the export apparatus [21]. Furthermore, a cross-complementation study showed that members of the InvA membrane protein family (which includes FlhA) could substitute to some degree functionally for *Salmonella* InvA in the needle complex when the genes or fusions to part of the genes for the paralogs MxiA or LcrD were introduced into an *invA* mutant [22].

Here we investigated the similarities and difference between the type III export apparatus systems of different species of bacteria by cross-complementation analysis focusing on the trans-membrane protein FlhB. We replaced the *S. typhimurium flhB* gene with the *flhB* gene from another species (*Aquifex aeolicus*) or the trans-membrane encoding region of the *flhB* gene was replaced with that of the *A. aeolicus flhB* gene to create a gene fusion. We sought to understand properties of the cell, which would increase the ability of the foreign proteins to function within the *Salmonella* flagellar system, and expected to see drastic changes because the FlhB proteins are from distantly related species of bacteria. Suppressor mutants with improved motility were isolated from the strain expressing the gene fusion. The suppressor mutations were identified and characterized. Some of the suppressor mutations also increased motility with another gene fusion containing the trans-membrane encoding region of the *flhB* gene from a different species of bacteria, *Bacillus subtilis*.

## Results

### Expression of *A. aeolicus* FlhB and of an *A. aeolicus* FlhB/*S. typhimurium* FlhB Chimera

**Figure 1:**
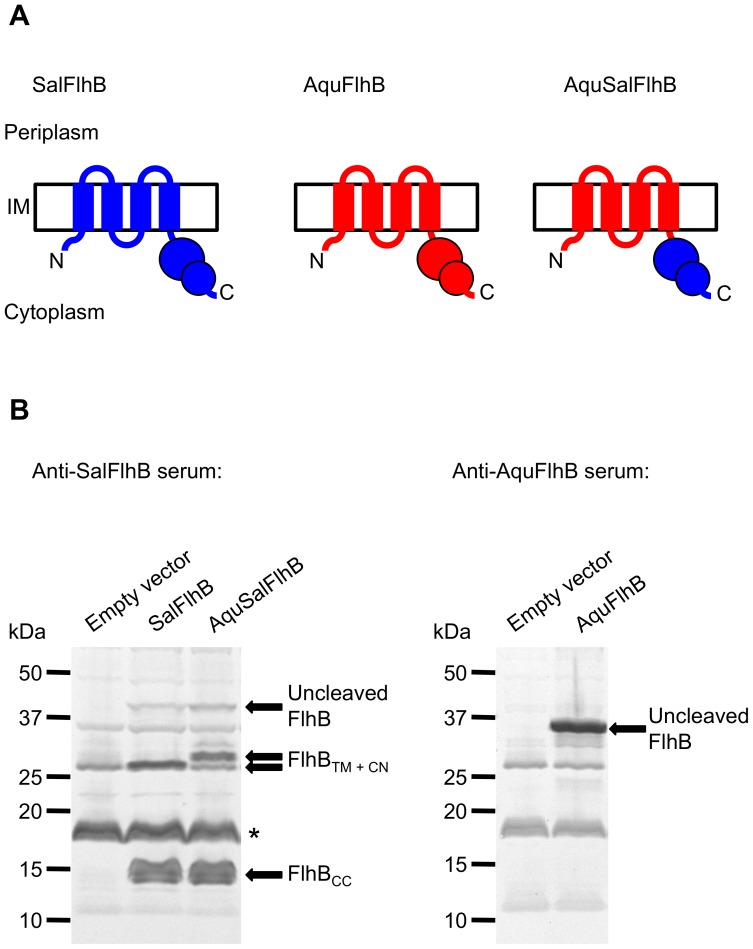
Affinity western blotting analysis of FlhB protein expression. (A) Cartoon representing the FlhB proteins expressed from plasmids. Sal, *S. typhimurium*, and Aqu, *A. aeolicus.* (B) Immunoblotting of FlhB proteins. The FlhB proteins were expressed in a Δ*flhB* null strain. FlhB naturally undergoes autocleavage into two fragments: The trans-membrane region and the N-terminus of the cytoplasmic domain (FlhB_TM + CN_); and the C-terminal region of the cytoplasmic domain (FlhB_CC_). The bands attributed to the FlhB proteins are labeled. The presence of a large non-specific band, which is also found in the lane loaded with lysate from cells containing empty plasmid vector is indicated with an asterisk. The FlhB_TM + CN_ fragment of AquSalFlhB (upper arrow) migrated more slowly than the FlhB_TM + CN_ fragment of SalFlhB (lower arrow).

In this study, a cross-complementation assay was performed focusing on the highly conserved and essential membrane protein component of the Type III export apparatus, FlhB. *A. aeolicus* is a deeply-rooted Gram-negative hyperthermophilic bacterium that possesses flagella and is evolutionarily very distant from *Salmonella* [23]. The FlhB protein of this species is among the most dissimilar to *S. typhimurium* FlhB at 32% identity. The FlhB protein from this species was chosen to begin this study because it is so distantly related to *S. typhimurium* FlhB, and it was interesting to see whether it would be functional. The FlhB protein of *S. typhimurium* (SalFlhB) was replaced with the FlhB protein of *A. aeolicus* (AquFlhB) or a chimera of the trans-membrane domain of *A. aeolicus* FlhB fused to the cytoplasmic domain of *Salmonella* FlhB (AquSalFlhB) (Figure 1A). The chimera was made to specifically investigate function of the trans-membrane region.

Whether AquFlhB or the AquSalFlhB chimera would be functional within the *S. typhimurium* flagellar system was interesting to determine. However, it was necessary to first determine whether the proteins could be made in *S. typhimurium*, especially considering there is a difference in codon bias between the species. The genes were introduced into a Δ*flhB* null *Salmonella* mutant. Affinity western blotting analysis with serum containing polyclonal antibodies specific for SalFlhB showed that the AquSalFlhB chimera and SalFlhB were expressed at a similar level (Figure 1B). FlhB naturally undergoes autocleavage within the cytoplasmic domain. For SalFlhB and the AquSalFlhB chimera, three fragments were anticipated and detected: Uncleaved FlhB; the FlhB trans-membrane region and N-terminal region of the cytoplasmic domain fragment; and also the C-terminal region of the cytoplasmic domain fragment. However, affinity western blotting analysis with serum containing polyclonal antibodies specific for AquFlhB identified only uncleaved wild-type AquFlhB. Autocleavage of this protein is much reduced within *Salmonella* cells at 37°C, perhaps due to enhanced thermostability or altered processing of the cytoplasmic domain under these conditions compared to SalFlhB.

**Figure 2:**
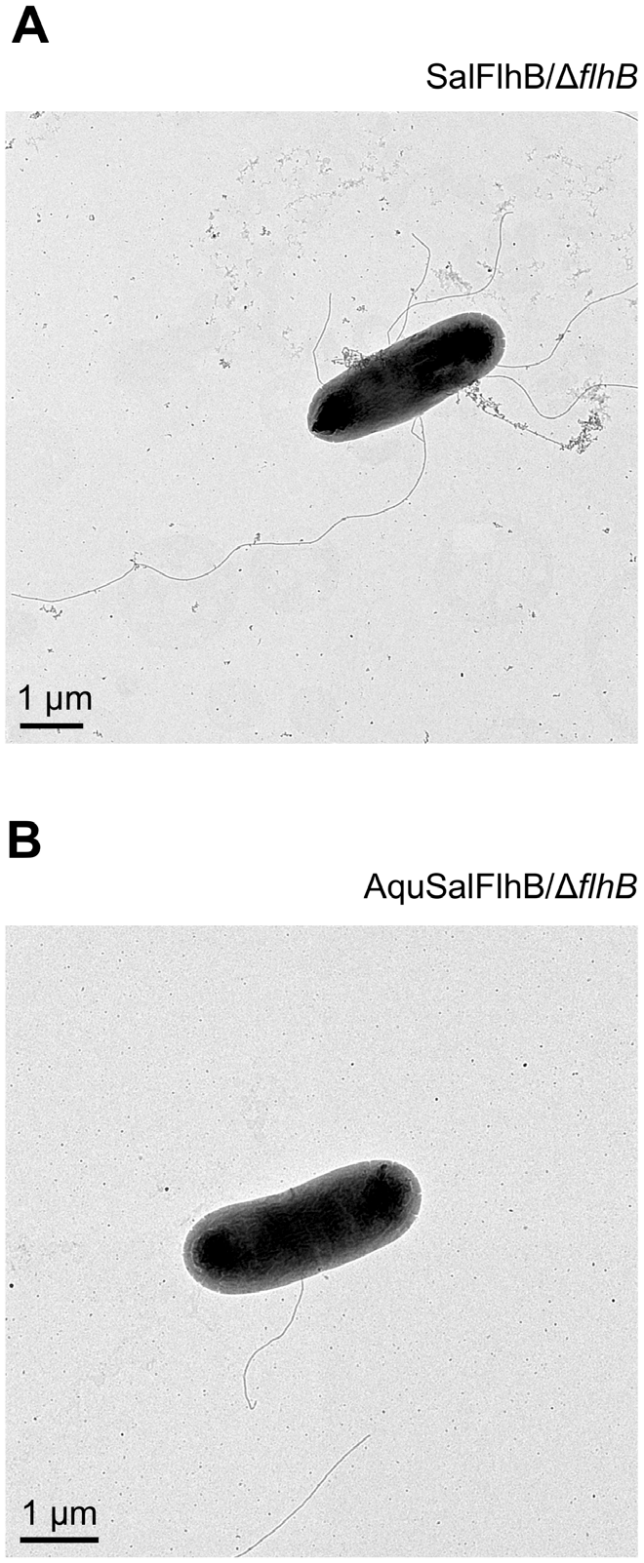
Cells expressing an AquSalFlhB chimera make rare flagella. (A) A *Salmonella* Δ*flhB* null mutant complemented with plasmid expressing wild-type *Salmonella* FlhB forming peritrichous flagella. (B) A Δ*flhB* mutant complemented with plasmid expressing an AquSalFlhB chimera forming a single flagellum. Representative images are shown. Cells were stained with 4% uranyl acetate.

The ability of the Δ*flhB* mutant to synthesize flagella with SalFlhB, AquFlhB or AquSalFlhB expressed from plasmids was examined by transmission electron microscopy. For Δ*flhB* mutant cells complemented with wild-type SalFlhB, all possessed greater than 6 flagella (Figure 2A). A sample of greater than 30 cells was examined for cells expressing AquFlhB, but the cells did not produce flagella. However, 13% of cells expressing AquSalFlhB formed one to two flagella (Figure 2B).

### Identification of Suppressor Mutations that Improved Motility for Cells Expressing the AquSalFlhB Chimera

**Figure 3:**
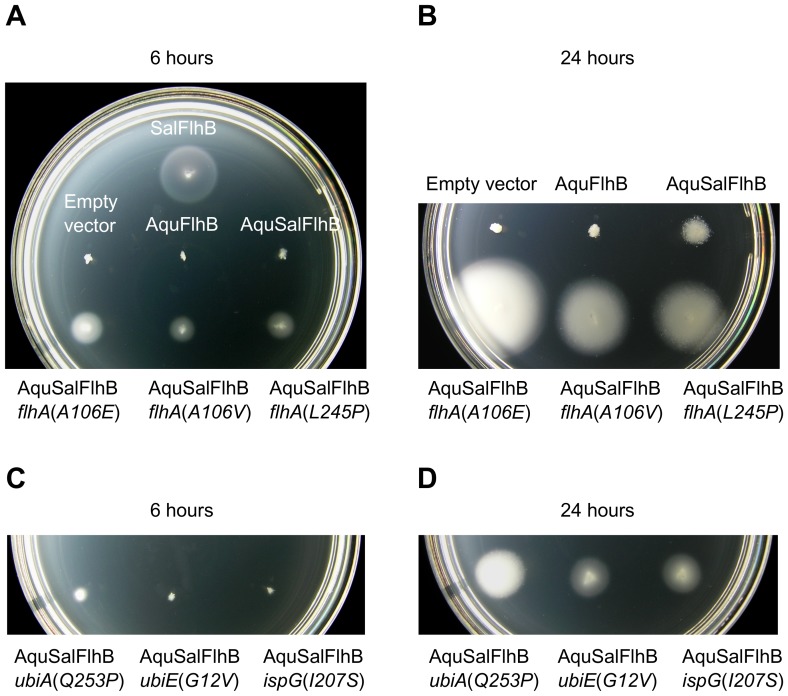
Extragenic suppressor mutations enhance complementation of motility by an AquSalFlhB chimera. All strains contained a Δ*flhB* null mutation, and strains containing additional suppressor mutations are labeled. Sal  =  *Salmonella* and Aqu  =  *A. aeolicus*. (A) Motility phenotype at 6 hours of Δ*flhB* strains carrying either empty plasmid vector, or carrying plasmid expressing SalFlhB, AquFlhB, or an AquSalFlhB chimera, with additional suppressor mutations in *flhA* as labeled. (B) Motility phenotype of the same strains as in panel A at 24 hours. The Δ*flhB* mutant complemented with SalFlhB was not included on this plate, because after 24 hours it would be overgrown. (C) Motility phenotype at 6 hours for Δ*flhB* strains with suppressor mutations in *ubiA*, *ubiE*, or *ispG* genes and expressing AquSalFlhB (D) Motility phenotype at 24 hours of the same strains as in panel C. The soft tryptone agar plates were incubated at 30°C.

*S. typhimurium* Δ*flhB* null mutant cells expressing the wild-type *A. aeolicus flhB* gene or the *flhB* fusion chimera gene from plasmids were inoculated into soft tryptone agar. After 48 hours, a weak zone of swimming was observed for both strains (data not shown). For the cells expressing the fusion *flhB* gene, suppressor mutants with enhanced motility were generated. No suppressor mutants recovered wild-type levels of motility. Six suppressor mutants with enhanced motility were isolated and their DNA purified. Initial sequencing of plasmid DNA demonstrated that the sequence of the fusion gene was unaltered, indicating that extragenic suppressor mutations were encoded on the chromosome. The suppressor mutations encoded within the genomes of the six strains were identified by whole genome sequence analysis. Each suppressor mutant strain encoded a single mutation that allowed rescue of motility (Table 1; Figure 3).

**Table 1:** Location of suppressor mutations that allowed an AquSalFlhB chimera to rescue motility of a Δ*flhB* mutant.

Strain	Chromosomal location^a^	Gene	Protein	Mutation
CB554	2009974 G to T	*flhA*	FlhA	Ala106Glu
CB555	2009974 G to A	*flhA*	FlhA	Ala106Val
CB556	2009557 A to G	*flhA*	FlhA	Leu245Pro
CB418	4455967 A to C	*ubiA*	4-hydroxybenzoate octaprenyltransferase	Gln253Pro
CB419	4177325 G to T	*ubiE*	ubiquinone/menaquinone biosynthesis methyltransferase	Gly12Val
CB420	2656755 A to C	*ispG* (*gcpE*)	4-hydroxy-3-methylbut-2-en-1-yl diphosphate synthase	Ile207Ser

a The chromosomal location refers to the position on the *S*. *typhimurium* str. LT2 chromosome, NCBI Reference Sequence: NC_003197.1. Mutations were identified by whole genome sequencing and confirmed by Sanger sequencing.

Out of these six strains, three bore missense mutations in the *flhA* gene, which encodes the export apparatus protein FlhA, at positions corresponding to A106E, A106V, or L245P. These mutations are all within predicted periplasmic loops of FlhA. Surprisingly, the three other suppressor mutations were found in non-flagellar genes involved in biosynthesis of ubiquinone (coenzyme Q; UQ). The first mutation was identified in *ubiA*, encoding 4-hydroxybenzoate octaprenyltransferase, mutated at position Q253P. The second mutation was in *ubiE*, encoding ubiquinone/menaquinone biosynthesis methyltransferase, mutated at position G12V. The third mutation was found in *ispG* (also known as *gcpE*), encoding 4-hydroxy-3-methylbut-2-en-1-yl diphosphate synthase, mutated at position I207S.

**Figure 4:**
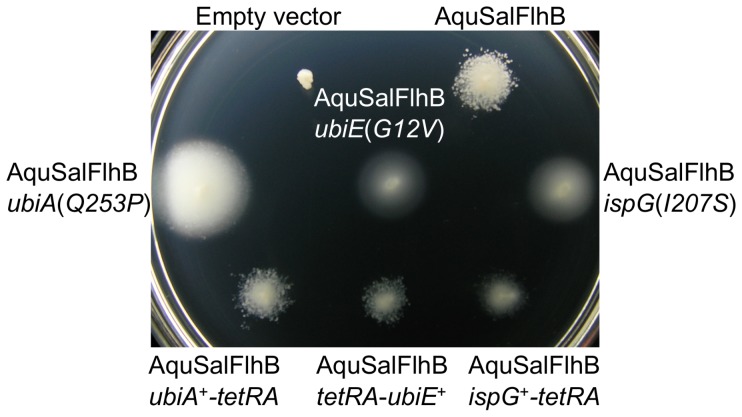
Restoring the *ubiA*(*Q253P*), *ubiE*(*G12V*), or *ispG*(*I207S*) alleles with wild-type genes abolished suppression. The AquSalFlhB chimera was expressed from plasmid vector. All strains contained a Δ*flhB* null mutation, and strains containing additional mutations are labeled. The *ubiA*(*Q253P*), *ubiE*(*G12V*), or *ispG*(*I207S*) alleles were replaced with wild-type genes in recipient strains by P22-mediated transduction, using donor strains with a *tetRA* (Tet^R^) cassette engineered on the chromosome to a site adjacent to the target gene. The soft tryptone agar plate was incubated at 30°C for 24 hours.

The respiratory chain of *Salmonella* utilizes three different types of quinones with different redox potentials as electron carriers: ubiquinone, menaquinone (vitamin K_2_) and demethylmenaquinone [24]. Ubiquinone and demethylmenaquinone/menaquinone are synthesized by independent pathways, which are coupled by the substrate chorismate. The enzyme 4-hydroxybenzoate octaprenyltransferase is part of the ubiquinone biosynthesis pathway, and ubiquinone/menaquinone biosynthesis methyltransferase is for ubiquinone and menaquinone biosynthesis. The *ispG* gene is essential for cell survival and 4-hydroxy-3-methylbut-2-en-1-yl diphosphate synthase is part of the mevalonate-independent pathway for isoprenoid biosynthesis, which has many uses for the cell and is used to synthesize octaprenyl diphosphate for quinone biosynthesis [24], [25]. Since the *ubiA*, *ubiE*, and *ispG* genes are not flagellar genes it was confirmed that the mutations in these genes were responsible for enabling the chimera to rescue motility of the Δ*flhB* mutant by restoring these genes in *cis* to wild-type. The *ubiA*(*Q253P*), *ubiE*(*G12V*), and *ispG*(*I207S*) alleles were replaced with wild-type genes linked to a *tetRA* tetracycline-resistance gene cassette using P22 bacteriophage-mediated transduction, and the enhanced motility conferred on cells expressing the *flhB* fusion gene was lost (Figure 4). None of the six suppressor mutations improved motility with cells producing AquFlhB (data not shown).

### The Suppressor Mutations Improved Biogenesis of Flagella and Motility for Cells Expressing the AquSalFlhB Chimera

A small increase in motility was observed in soft tryptone agar for the strains bearing suppressor mutations and expressing the AquSalFlhB chimera. Transmission electron microscopy was used to examine if there was also an increase in the numbers of flagella for the cells. Cells producing wild-type *S. typhimurium* FlhB possessed from 4 to greater than 10 flagella per cell (Table 2). For the Δ*flhB* mutant expressing the AquSalFlhB chimera, 13% of cells had one or two flagella. The *flhA*(*A106E*), *flhA*(*A106V*), *flhA*(*L245P*), *ubiA*(*Q253P*), *ubiE*(*G12V*), or *ispG*(*I207S*) suppressors enhanced both the proportion of cells synthesizing flagella (between 38–60%) and the numbers of flagella per cell (1 to 4) for cells expressing AquSalFlhB. A similar proportion of motile cells were observed when the cultures were examined by phase contrast microscopy (data not shown). No flagella or motile cells were observed by microscopy when Δ*flhB* mutant cells harbored empty plasmid vector or vector carrying the *A. aeolicus flhB* gene.

**Table 2:** Measurement of flagella numbers.

Strain^a^	Cells with flagella (%)b,c	Range of flagella numbersb,c,d
Wild-type, *flhB* ^+^	100	4– >10
Empty plasmid/Δ*flhB*	0	0
SalFlhB/Δ*flhB*	100	6– >10
AquFlhB/Δ*flhB*	0	0
AquSalFlhB/Δ*flhB*	13	1–2
AquSalFlhB/Δ*flhB ubiA*(*Q253P*)	48	1–4
AquSalFlhB/Δ*flhB ubiE*(*G12V*)	60	1–4
AquSalFlhB/Δ*flhB ispG*(*I207S*)	52	1–3
AquSalFlhB/Δ*flhB flhA*(*A106E*)	43	1–3
AquSalFlhB/Δ*flhB flhA*(*A106V*)	47	1–4
AquSalFlhB/Δ*flhB flhA*(*L245P*)	38	1–2

a FlhB proteins were expressed from plasmid vector.

b At least 30 cells were observed for each strain.

c Cultures were harvested at mid-exponential phase, OD_600_ 0.6.

d Some strains made more than 10 flagella per cell.

### The *flhA*(*A106E*), *flhA*(*A106V*), and *flhA*(*L245P*) Suppressors are Gain-of-function Mutations

**Figure 5:**
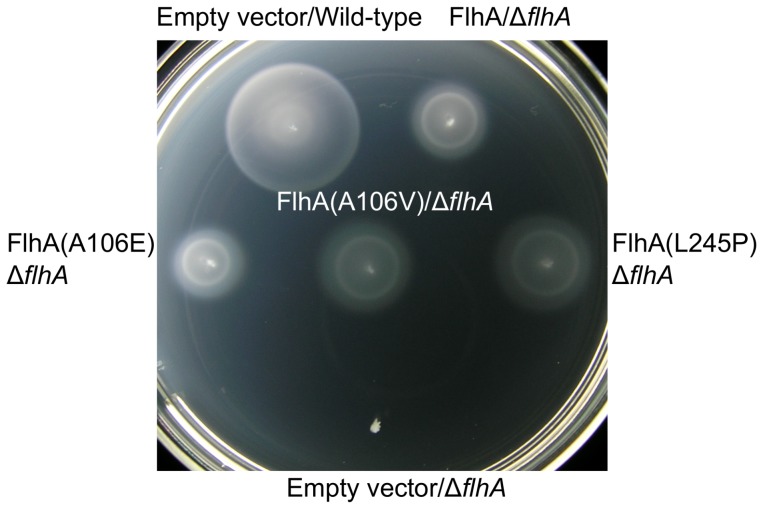
FlhA(A106E), FlhA(A106V), or FlhA(L245P) complement motility of a Δ***flhA*** **mutant.** Wild-type *Salmonella* containing empty plasmid vector, or strains containing a Δ*flhA*::FRT null mutation complemented by plasmid expressing wild-type FlhA or expressing FlhA mutants, were inoculated into soft tryptone agar. The Δ*flhA*::FRT mutant containing empty plasmid vector was inoculated as a negative control. The soft tryptone agar plate was incubated at 30°C for 5.5 hours.

The mechanism of suppression by the mutations in the *flhA* gene was investigated further: Plasmids carrying either the wild-type *flhA* gene, *flhA*(*A106E*), *flhA*(*A106V*), or *flhA*(*L245P*) were transformed into a Δ*flhA*::FRT null mutant, and the transformants inoculate into soft tryptone agar to examine motility (Figure 5). This was to determine if the FlhA mutants could still function effectively within the otherwise wild-type *Salmonella* flagellar export apparatus. The Δ*flhA* mutant complemented with plasmid expressing wild-type FlhA was slightly less motile than wild-type *S. typhimurium* containing empty plasmid vector. This indicated that the FlhA protein was over-expressed from the plasmid vector. Nevertheless, plasmids expressing the FlhA missense mutants complemented the Δ*flhA* mutant for motility to a similar degree as wild-type FlhA. This suggested that *flhA*(*A106E*), *flhA*(*A106V*) and *flhA*(*L245P*) were gain-of-function mutations.

### The *flhA*(*A106E*), *flhA*(*A106V*), and *flhA*(*L245P*) Suppressors also Improved Motility with a *B. subtilis* FlhB/*S. typhimurium* FlhB Chimera

The FlhA(A106E), FlhA(A106V), and FlhA(L245P) mutants have been shown to allow improved function of the AquSalFlhB chimera within the *Salmonella* type III export apparatus. They have also been demonstrated to function effectively within the otherwise wild-type *Salmonella* flagellum. This raised the question whether the mechanism of suppression was specific to the AquSalFlhB chimera, and in particular the trans-membrane domain from *A*. *aeolicus* FlhB. Consequently, whether the mutations within the *flhA* gene could increase motility of cells expressing another FlhB ortholog was investigated. FlhB from the Gram-positive bacterium *Bacillus subtilis* (BacFlhB) was selected, because it is also distantly related to SalFlhB with an identity of 36%. In comparison, the identity between SalFlhB and AquFlhB is 32%, and the identity between AquFlhB and BacFlhB is 37%. SalFlhB, AquFlhB, and BacFlhB can each be aligned over their length (Figure S1). A gene fusion encoding a chimera of the trans-membrane region of BacFlhB fused to the cytoplasmic domain of SalFlhB, to express a BacSalFlhB chimera was also made (Figure S2). For the BacSalFlhB chimera: uncleaved FlhB, the FlhB trans-membrane region and N-terminal region of the cytoplasmic domain fragment, and also the C-terminal region of the cytoplasmic domain fragment could be detected by immunoblotting with serum containing polyclonal anti-SalFlhB antibodies. For Δ*flhB* mutant cells expressing BacSalFlhB, 33% formed 1 or 2 flagella (Figure S2). However, no flagella were observed for Δ*flhB* mutant cells expressing BacFlhB.

**Figure 6:**
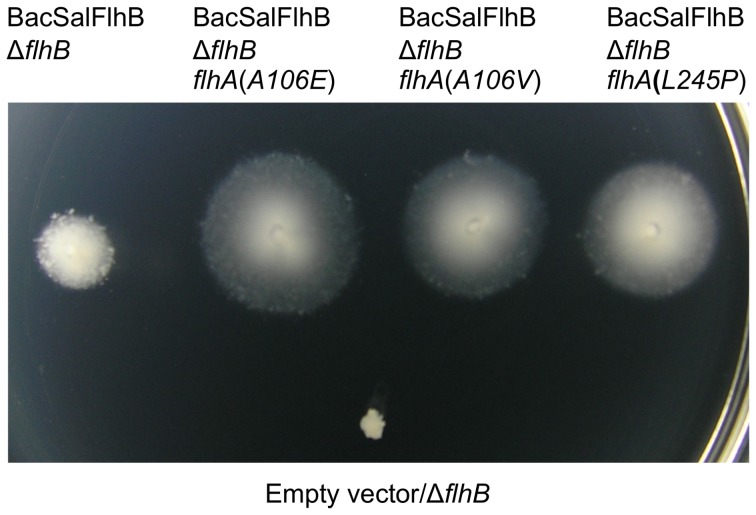
Suppressor mutations in the *flhA* gene enhance complementation of motility by BacSalFlhB. Motility in soft tryptone agar for strains containing a Δ*flhB* null mutation, or the Δ*flhB* mutation with suppressor mutations in the *flhA* gene, and complemented with plasmid expressing a BacSalFlhB chimera. The chimera was made of the trans-membrane domain from *B. subtilis* FlhB fused to the cytoplasmic domain from *S. typhimurium* FlhB. The Δ*flhB* mutant containing empty plasmid vector was inoculated as a negative control. The soft tryptone agar plate was incubated for 20 hours at 30°C.

The suppressor mutations in the *flhA* gene all improved motility of Δ*flhB* mutant cells expressing the BacSalFlhB chimera in soft tryptone agar (Figure 6). This demonstrated that the mechanism of suppression was not specific to the AquSalFlhB chimera. The *flhA*(*A106E*), *flhA*(*A106V*), or *flhA*(*L245P*) mutations did not improve motility for cells carrying the wild-type *B. subtilis flhB* gene (data not shown). Also, the suppressor mutations in the genes for ubiquinone biosynthesis could not enhance motility for cells complemented with the wild-type *B. subtilis flhB* gene or with the *B. subtilis*/*S. typhimurium flhB* fusion gene (data not shown).

### The *ubiA*(*Q253P*), *ubiE*(*G12V*) and *ispG*(*I207S*) Mutations Severely Reduced Motility in Soft-tryptone Agar with Wild-type FlhB, but Flagellar Biogenesis was Less Reduced

**Figure 7:**
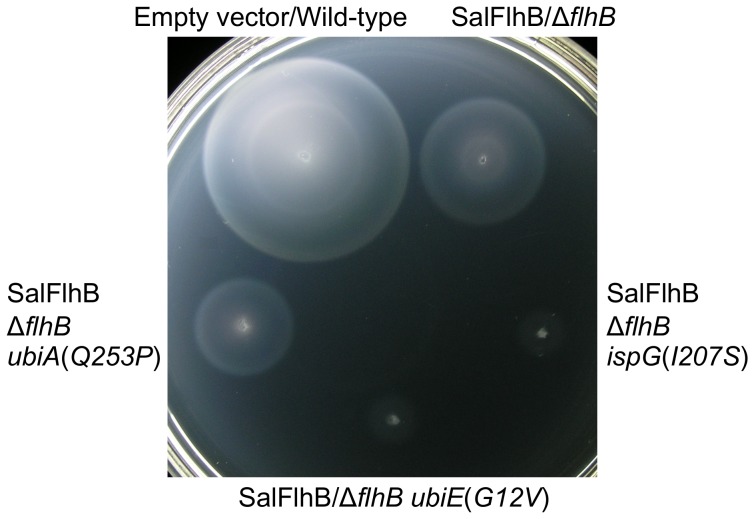
Motility of strains bearing mutations in genes for ubiquinone biosynthesis complemented by SalFlhB. Motility in soft tryptone agar for wild-type *Salmonella* containing empty plasmid vector, or for strains containing a Δ*flhB* null mutation, or the Δ*flhB* mutation with suppressor mutations in the *ubiA*(*Q253P*), *ubiE*(*G12V*) or *ispG*(*I207S*) genes complemented by plasmid carrying the wild-type *Salmonella flhB* gene. The soft tryptone agar plate was incubated for 7.5 hours at 30°C.

Whether the *ubiA*(*Q253P*), *ubiE*(*G12V*) or *ispG*(*I207S*) alleles would affect motility of cells expressing wild-type *S. typhimurium* FlhB was examined. It was not possible to introduce the mutated *ubiA*(*Q253P*), *ubiE*(*G12V*) or *ispG*(*I207S*) alleles into a wild-type *flhB* ^+^ background, because growth rate of strains bearing these mutations was markedly reduced and genetic manipulation of strains was consequently difficult. This indicated that the activity of the respiratory chain was compromised for strains bearing these mutations. Therefore, motility in soft tryptone agar was examined for strains bearing a Δ*flhB* mutation with or without the *ubiA*(*Q253P*), *ubiE*(*G12V*) or *ispG*(*I207S*) alleles and containing plasmid carrying the wild-type *flhB* gene. Motility of cells bearing these mutations and expressing *S. typhimurium* FlhB was drastically reduced in soft tryptone agar (Figure 7). However, motility in soft tryptone agar is affected by numerous properties that are reliant on the proton motive force and an active respiratory chain, such as cell division time, activity of the flagellar motor, as well as the rate of flagellar biogenesis. Therefore, the presence of flagella was examined directly for cells expressing *S. typhimurium* FlhB by transmission electron microscopy. For cells with the Δ*flhB* mutation complemented with *S. typhimurium* FlhB, 100% formed more than 6 flagella. For *ubiA*(*Q253P*) cells, 50% formed between 1 to 8 flagella; for *ubiE*(*G12V*) cells, 59% formed between 1 to 8 flagella; and for *ispG*(*I207S*) cells, 82% formed between 1 to 8 flagella. A similar proportion of cells were estimated to be motile when the cultures were examined by phase contrast microscopy, as were observed to possess flagella when examined by electron microscopy (data not shown).

### The *ubiA*(*Q253P*), *ubiE*(*G12V*) and *ispG*(*I207S*) Alleles are Loss-of-function Mutations, which Reduced the Synthesis of Ubiquinone

The enzyme products of the *ubiA*, *ubiE*, and *ispG* genes are required for the biosynthesis of the electron-carrier ubiquinone. The consequences of the *ubiA*(*Q253P*), *ubiE*(*G12V*) or *ispG*(*I207S*) mutations on the quinone pool of the cytoplasmic membrane was examined for the strains by reversed-phase high-performance liquid chromatography (Table 3). The membrane quinone pool was analyzed for the strains grown aerobically to mid-exponential phase (OD_600_ 0.6) in Luria Broth, because this phase of growth is optimal for flagella biogenesis.

**Table 3:** Quantification of the quinone pool of suppressor strains.

Straina,b	Amount of ubiquinone-8 (ng/mg of cell protein)^c^
Wild-type, *flhB* ^+^	780±90
Δ*flhB*	910±150
Empty plasmid/Δ*flhB*	700±70
SalFlhB/Δ*flhB*	920±170
AquSalFlhB/Δ*flhB*	850±150
AquSalFlhB/Δ*flhB ubiA*(*Q253P*)	110±70
AquSalFlhB/Δ*flhB ubiE*(*G12V*)	N.D.^d^
AquSalFlhB/Δ*flhB ispG*(*I207S*)	60±30

a FlhB proteins were expressed from plasmid vector.

b Cultures were harvested at mid-exponential phase, OD_600_ 0.6.

c Standard deviations are shown for measurements performed in triplicate.

d N.D., None detected. Ubiquinone-8 was not detected in cells bearing a *ubiE*(*G12V*) mutation, however, 2-octaprenyl-6-methoxy-1,4-benzoquinone (480±150 ng/mg of cell protein) was detected.

*Salmonella* ubiquinone has eight isoprenoid units. For cells bearing a *ubiA*(*Q253P*) allele, the pool of ubiquinone-8 was reduced by at least 6-fold. Wild-type *ubi* ^+^ cells made about 700–920 ng ubiquinone/mg of cell protein, while *ubiA*(*Q253P*) cells made about 110 ng ubiquinone/mg of protein. For cells bearing the *ispG*(*I207S*) mutation, the ubiquinone concentration was reduced further to 60 ng/mg of protein. While for *ubiE*(*G12V*) cells no ubiquinone was detected at a limit of detection of 40 ng ubiquinone/mg of cell protein. Moreover, *ubiE*(*G12V*) cells accumulated 2-octaprenyl-6-methoxy-1,4-benzoquinone to 480 ng/mg of cell protein, and this is the substrate for ubiquinone/menaquinone biosynthesis methyltransferase, which is the enzyme product of the *ubiE* gene. Therefore, these mutations had a negative effect on the enzymes encoded by these genes and the consequence was a reduced level of ubiquinone for the respiratory chain.

### Restoring Ubiquinone Biosynthesis for Δ*flhB ubiA*(*Q253P*) Cells by Addition of Exogenous 4-hydroxybenzoate Reduced Flagella Biogenesis with AquSalFlhB

**Figure 8:**
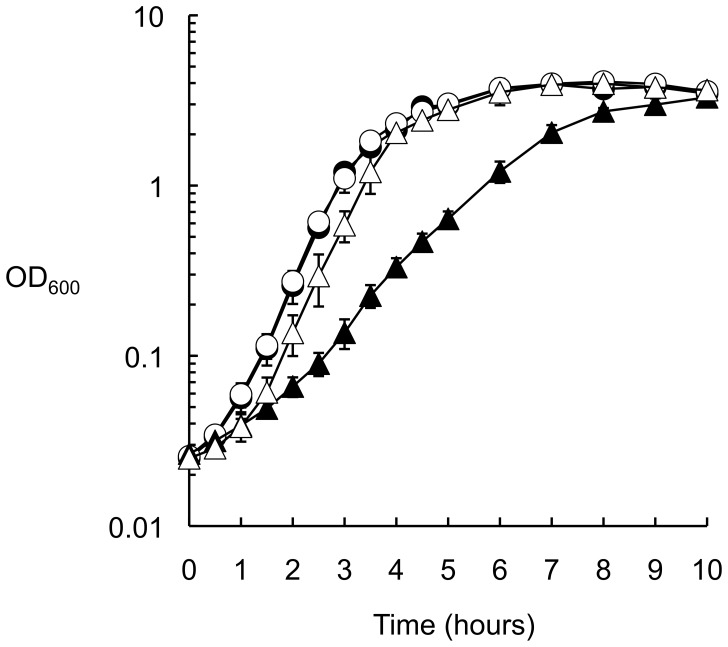
Exogenous 4-hydroxybenzoate restores growth of a strain bearing a *ubiA*(Q253P) mutation. Strains were grown aerobically at 37°C in Luria Broth supplemented with 4-hydroxybenzoate as required and sampled at intervals. • Δ*flhB* mutant, ○ Δ*flhB* mutant with 1 mM 4-hydroxybenzoate, ▴ Δ*flhB ubiA*(*Q253P*) mutant, ▵ Δ*flhB ubiA*(*Q253P*) mutant with 1 mM 4-hydroxybenzoate.

In the pathway for ubiquinone biosynthesis, the enzyme product of the *ubiA* gene, 4-hydroxybenzoate octaprenyltransferase, catalyzes the conversion of 4-hydroxybenzoate to 3-octaprenyl-4-hydroxybenzoate. In an early study of ubiquinone biosynthesis, it was found that the reduced growth of some *ubiA* mutant strains of *E. coli* could be restored if excess 4-hydroxybenzoate (greater than 0.1 mM) was added exogenously to the growth medium [26]. Here, the effect of 1 mM sodium 4-hydroxybenzoate on the growth of *S. typhimurium* strains with or without the *ubiA*(*Q253P*) allele was examined for cells grown aerobically in Luria Broth. The addition of 4-hydroxybenzoate did not affect growth of wild-type *ubi* ^+^ cells, so 4-hydroxybenzoate was not toxic at this concentration. Growth was markedly reduced for cells bearing the *ubiA*(*Q253P*) allele, but the addition of 4-hydroxybenzoate clearly recovered growth to near wild-type (Figure 8). Following this the quinone pool of the cell was measured (Table 4). It was found that cells bearing the *ubiA*(*Q253P*) mutation produced 10% of the amount ubiquinone of wild-type *ubi* ^+^ cells in exponential phase or in stationary phase, and the addition of 4-hydroxybenzoate recovered ubiquinone biosynthesis to 50% of the normal level for cells in exponential phase and 30% of the normal level for cells grown to stationary phase. For stationary phase grown cells, menaquinone was also detected. Cells bearing the *ubiA*(*Q253P*) mutation accumulated 40% the normal level of menaquinone, which could be recovered to wild-type levels by the addition of 4-hydroxybenzoate.

**Table 4:** Exogenous 4-hydroxybenzoate can restore the quinone pool for cells bearing a *ubiA*(*Q253P*) mutation.

Strain^a^	Growth phase^b^	4-hydroxybenzoate (1 mM)	Quinone^c^	Amount of quinone (ng/mg cell protein)^d^
*ubi* ^+^	Exponential	−	UQ-8	930±160
		+	UQ-8	930±40
*ΔubiA*(*Q253P*)	Exponential	−	UQ-8	90±70
		+	UQ-8	500±20
*ubi* ^+^	Stationary	−	UQ-8	1750±180
		+	UQ-8	1950±240
*ubiA*(*Q253P*)	Stationary	−	UQ-8	160±90
		+	UQ-8	590±30
*ubi* ^+^	Stationary	−	MK-8	230±20
		+	MK-8	210±40
*ubiA*(*Q253P*)	Stationary	−	MK-8	90±20
		+	MK-8	210±20

a Both strains bore Δ*flhB* mutations.

b For exponential phase, cells were harvested at OD_600_ 0.6.

c For cells in stationary phase, ubiquinone-8 (UQ-8) and menaquinone-8 (MK-8) were detected.

d Standard deviations are shown for measurements performed in triplicate.

**Figure 9:**
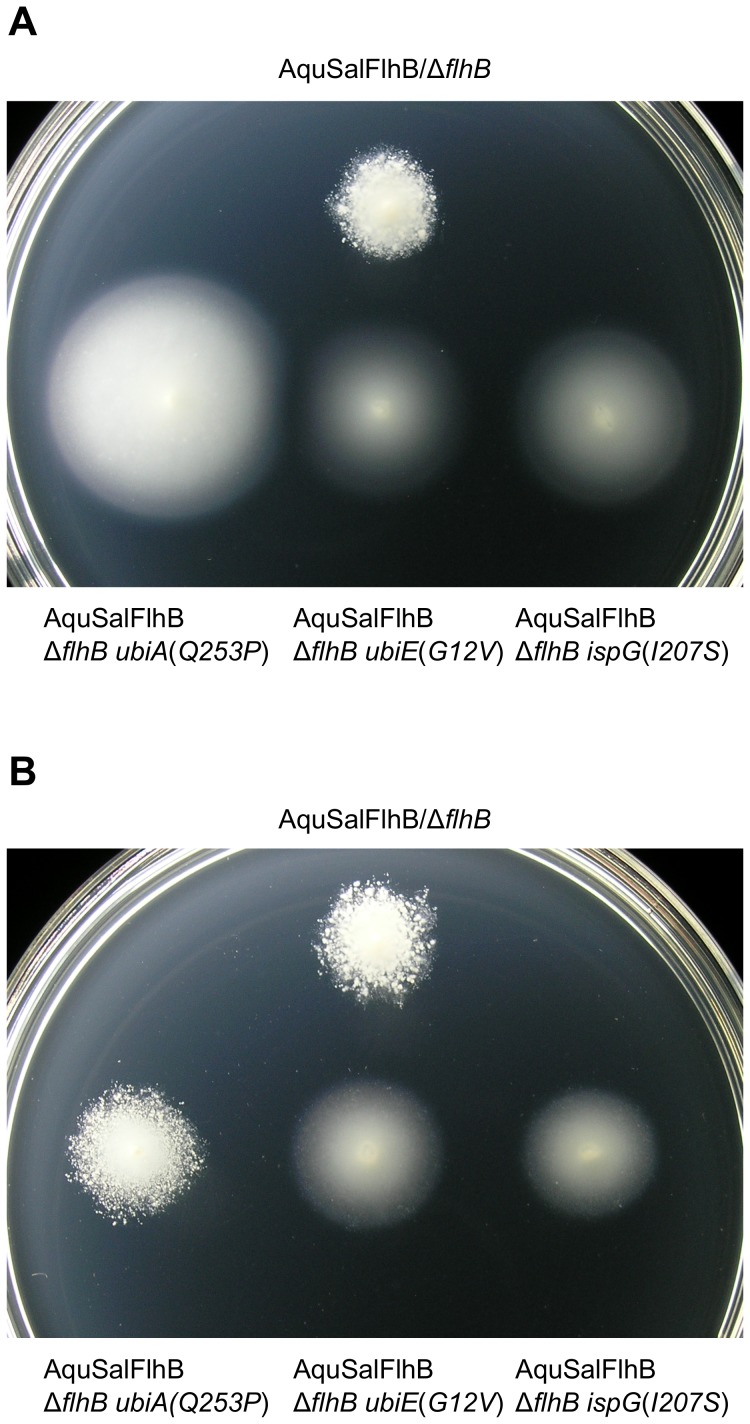
Recovery of ubiquinone biosynthesis for the *ubiA*(*Q253P*) mutant reduces complementation of motility by AquSalFlhB. (A) Motility in soft tryptone agar for strains containing a Δ*flhB* null mutation, or the Δ*flhB* mutation with suppressor mutations in the *ubiA*(*Q253P*), *ubiE*(*G12V*) or *ispG*(*I207S*) genes complemented by plasmid expressing the AquSalFlhB chimera. (B) Motility for the same strains as in panel A, inoculated into soft tryptone agar containing 1 mM 4-hydroxybenzoate. The exogenous 4-hydroxybenzoate restored ubiquinone biosynthesis for the strain bearing the *ubiA*(*Q253P*) mutation. The soft tryptone agar plates were incubated for 33 hours at 30°C.

The effect on motility of adding 1 mM 4-hydroxybenzoate to soft tryptone agar for cells bearing a Δ*flhB* mutation expressing AquSalFlhB with or without the *ubiA*(*Q253P*), *ubiE*(*G12V*), or *ispG*(*I207S*) suppressor mutations was examined. This was done to test whether restoring the ubiquinone pool for the strain bearing the *ubiA*(*Q253P*) allele would abolish the enhancement of motility with AquSalFlhB. Swimming motility of cells bearing the *ubiA*(*Q253P*) mutation and expressing AquSalFlhB was clearly reduced when the membrane pool of ubiquinone was restored by adding exogenous 4-hydroxybenzoate to the soft tryptone agar (Figure 9). When the cells were examined by transmission electron microscopy, numbers of flagella for the Δ*flhB ubiA*(*Q253P*) strain expressing the AquSalFlhB chimera were also reduced when the strain was grown with 1 mM 4-hydroxybenzoate, from 48% of cells possessing 1 to 4 flagella to 20% of cells possessing 1 to 2 flagella. The cells were also less motile when the growth medium was supplemented with 4-hydroxybenzoate and examined by phase contrast microscopy (data not shown).

## Discussion

In this study, a cross-complementation analysis was performed with the type III export apparatus protein FlhB to investigate the level of conservation between different type III export apparatuses, and the type of properties which could allow a host flagellar system to function better with a foreign homolog. An AquSalFlhB chimera and a BacSalFlhB chimera were expressed, which weakly complemented a *Salmonella* Δ*flhB* null mutant for the synthesis of flagella and motility. This showed that despite millions of years of separation between the species the trans-membrane domains of FlhB have maintained a similar functional mechanism. Suppressor mutants with enhanced motility were isolated, and the type of suppressor mutation that increased the ability of AquSalFlhB to function within the *Salmonella* flagellar system could be categorized into two groups: The first group of suppressor mutations were found in the *flhA* gene, and the second group of mutations were localized in genes required for the biosynthesis of the respiratory chain electron-carrier ubiquinone.

The mutations in the *flhA* gene, which enabled the *Salmonella* export apparatus to function better with the AquSalFlhB chimera, *flhA*(*A106E*), *flhA*(*A106V*) and *flhA*(*L245P*) are all within regions encoding for predicted periplasmic loops of FlhA (Figure S3). The mutations also increased the ability of the export apparatus to function with the BacSalFlhB chimera. Since, the FlhA(A106E), FlhA(A106V), and FlhA(L245P) mutants were able to complement a strain bearing a Δ*flhA*::FRT null mutation for motility to a similar level as wild-type FlhA, it suggests that the mutations are gain-of-function mutations. Position Leu245 is very close to Asp249, which has recently been shown to be critical for FlhA function, demonstrating that this region of FlhA is very important in function [27]. The mechanism of these suppressors should rescue how the trans-membrane regions of *A. aeolicus* FlhB and *S. typhimurium* FlhB differ most in their interactions with other cellular components. However, these mutations do not appear to have made *S. typhimurium* FlhA more like *A. aeolicus* FlhA: The N-terminal trans-membrane regions of *S. typhimurium* FlhA, *B. subtilis* FlhA, and *A. aeolicus* FlhA are highly conserved. For instance, Leu245 of *S*. *typhimurium* is fully conserved in an alignment of the FlhA proteins (Figure S3). It has previously been demonstrated that the cytoplasmic domains of FlhA and FlhB interact [12], [28]. FlhA and FlhB must therefore be in close proximity with each other. It is possible that these suppressors could have increased the level of protein-protein interaction between FlhA and FlhB.

The suppressor mutations in the *ubiA*, *ubiE* or *ispG* genes were surprising. Over 30 years ago, it was observed that *E. coli* bearing mutations in *ubiA*, *ubiD*, *ubiB*, or *ubiG* genes of the ubiquinone biosynthesis pathway grown aerobically and impaired in ubiquinone biosynthesis were immotile and lacked flagella [29], [30]. This is reasonable because ATP synthesis, flagella motor rotation and type III export are all required for motility, and all depend on the proton motive force generated by an active respiratory chain. Here, cells bearing either the *ubiA*(*Q253P*), *ubiE*(*Q253P*), or *ispG*(*I207S*) mutations made less ubiquinone in exponential phase revealing that they were loss-of-function mutations. However, it was found that despite reduced biosynthesis of ubiquinone these mutations also increased numbers of flagella for cells expressing the AquSalFlhB chimera. Adding excess 4-hydroxybenzoate to the growth medium recovered quinone biosynthesis for cells bearing the *ubiA*(*Q253P*) mutation, and this also reduced complementation of motility by the AquSalFlhB chimera. This suggests that it was the effect on the quinone pool rather than the mutations in the proteins themselves, which was important in the mechanism of suppression. At this point, it is not possible to say whether it was a reduced level of ubiquinone in the membrane or the effect on the respiratory chain, which led to increased motility with the AquSalFlhB chimera and is an area for future investigation.

This study has shown that screening for suppressor mutants with enhanced motility in soft tryptone agar from strains with a mutated flagellar export apparatus can identify interesting mutations within enzymes of the respiratory chain. To our knowledge, the three-dimensional structure for 4-hydroxybenzoate octaprenyltransferase has not yet been solved. It is predicted to be a trans-membrane protein, which consists of about seven trans-membrane α-helices (Figure S4). Gln253 is predicted to be located within trans-membrane α-helix number 6, and since the Q253P suppressor mutation resulted in a proline substitution it suggests that the α-helical structure might have been disrupted. The physiological defects attributed to this mutation could be bypassed by excess 4-hydroxybenzoate, which is the substrate for this enzyme and suggests that this mutant has a reduced affinity for 4-hydroxybenzoate.

Apparently, the crystal structure of ubiquinone/menaquinone biosynthesis methyltransferase has also not been solved. Although the crystal structures of the homologs Protein Isoaspartyl Methyltransferase and of the rebeccamycin 4′-*O*-methyltransferase, RebM have been reported, the N-terminal region of ubiquinone/menaquinone biosynthesis methyltransferase is unfortunately not conserved between the three proteins [31], [32]. The *ubiE*(*G12V*) mutation resulted in reduced ubiquinone biosynthesis and the 2-octaprenyl-6-methoxy-1,4-benzoquinone substrate accumulated, which suggests that this mutation inhibited the activity or reduced the level of the enzyme.

The crystal structures of two homologs of *Salmonella* 4-hydroxy-3-methylbut-2-en-1-yl diphosphate synthase (IspG) have been solved. The first one from *A. aeolicus*, PDB code 3NOY [33], has 45% identity with *S*. *typhimurium*, and the second from *Thermus thermophilus*, PDB code 2Y0F [34] has 36% identity. Both proteins have a very similar three-dimensional structure. The structure of IspG is made of two domains: An N-terminal domain that consists of a TIM barrel, and a C-terminal domain that has an αβ fold. A suppressor mutation was identified in *S. typhimurium* IspG at position Ile207. The affect of the mutation to this enzyme must be partial, since a complete gene knockout of *ispG* cannot be isolated because it is essential for cell viability [25]. The amino acid residue Ile207 of *S. typhimurium* is not conserved through the IspG family. It corresponds to Ile202 in *A. aeolicus* and Leu230 in *T. thermophilus*. This Ile residue comes just before two very well conserved residues, Thr-Glu, and is located in the loop that links two α-helices involved in IspG dimerization. Although Ile207 does not seem to take part in the function of IspG, the mutation I207S that we find in this study is likely to disturb the packing of these two α–helices and to change the protein surface polarity. These changes appear to have affected the function of IspG, and reduced the synthesis of quinones for the *ispG*(*I207S*) suppressor mutant.

## Materials and Methods

### Strains, Plasmids, and Growth Conditions

The bacterial strains and plasmids used in this study are listed in Table 5. *Salmonella* and *E. coli* strains were routinely cultured in aerobically in Luria Broth (LB) with continuous shaking (220 r.p.m.) at 37°C. Ampicillin was used in media at 100 µg ml^−1^ for *Salmonella* strains and 50 µg ml^−1^ for *E. coli* strains. Tetracycline was used at 15 µg ml^−1^ and kanamycin was used at 50 µg ml^−1^, where appropriate. Sodium 4-hydroxybenzoate was obtained from Tokyo Chemical Industry Co., Ltd. All other chemicals were obtained from Sigma-Aldrich (USA) or Wako (Japan).

**Table 5:** Strains and plasmids used in this study.

Strain or plasmid	Genotype or relevant characteristic	Source or reference
*Escherichia coli*		
DH5α	Recipient for cloning experiments	Invitrogen
BW25113	Plasmid pKD46 (Amp^R^)	35; CGSC7739^a^
*Salmonella enterica* serovar Typhimurium		
JR501	R^−^m^+^ for converting plasmids to *Salmonella* compatibility	42
TT13206	LT7 *phoN51*::Tn*10*-*11*(Tet^R^)	36; SGSC3718^b^
SJW1103	Wild-type for motility and chemotaxis	38
MKM50	Δ*flhB* null mutant	15
CB372	Δ*flhA22274*::FRT	This study
CB418	Δ*flhB ubiA141*(*Q253P*)	This study
CB419	Δ*flhB ubiE142*(*G12V*)	This study
CB420	Δ*flhB ispG1*(*I207S*)	This study
CB421	Δ*flhB ubiA* ^+^-*tetRA*	This study
CB422	Δ*flhB tetRA*-*ubiE* ^+^	This study
CB423	Δ*flhB ispG* ^+^-*tetRA*	This study
CB554	Δ*flhB flhA22279*(*A106E*)	This study
CB555	Δ*flhB flhA22280*(*A106V*)	This study
CB556	Δ*flhB flhA22283*(*L245P*)	This study
Plasmids		
pKD46	*λ*-Red genetic engineering plasmid; Amp^R^; temperature-sensitive *ori* (30°C)	35
pKD13	PCR template for isolation of an FRT-flanked kanamycin resistance cassette; Amp^R^ and Km^R^	35
pCP20	For Flp-catalyzed excision of the kanamycin-resistance cassette; Amp^R^ and Cam^R^; temperature-sensitive*ori* (30°C)	37
pTrc99A-FF4	Modified pTrc99A expression vector; Amp^R^	40
pMM26	pTrc99A-FF4 carrying wild-type *S. typhimurium flhB* (codons 1 to 383)	41
pMM130	pTrc99A-FF4 carrying wild-type *S. typhimurium flhA*	21
pTAB32	pTrc99A-FF4 carrying wild-type *A. aeolicus flhB* (codons 1 to 350)	This study
pTB333	pTrc99A-FF4 carrying a *flhB* fusion chimera gene consisting of *A. aeolicus flhB* (codons 1 to 212) fused to*S. typhimurium flhB* (codons 219 to 383)	This study
pCB531	pTrc99A-FF4 carrying wild-type *B. subtilis flhB* (codons 1 to 360)	This study
pCB532	pTrc99A-FF4 carrying a *flhB* fusion chimera gene consisting of *B. subtilis flhB* (codons 1 to 223) fused to*S. typhimurium flhB* (codons 219 to 383)	This study
pCB537	pTrc99A-FF4 carrying *S. typhimurium flhA*(*A106E*)	This study
pCB539	pTrc99A-FF4 carrying *S. typhimurium flhA*(*A106V*)	This study
pCB542	pTrc99A-FF4 carrying *S. typhimurium flhA*(*L245P*)	This study

a CGSC, *Escherichia coli* Genetic Stock Center, Yale University, USA.

b SGSC, *Salmonella* Genetic Stock Centre, University of Calgary, Canada.

### Construction of Strains and Plasmids

Oligonucleotides used to construct strains and plasmids are provided in Table S1. To introduce a tetracycline-resistance cassette onto the chromosome of target strains, bacteriophage *λ*-Red-based recombination was used employing plasmid pKD46 [35]. Tetracycline-resistance cassettes were obtained by PCR using strain TT13206 genomic DNA as template [36]. The Δ*flhA*::FRT null mutation was made using a kanamycin cassette obtained by PCR from plasmid pKD13, which was inserted onto the chromosome using plasmid pKD46, and the kanamycin cassette was resolved using plasmid pCP20 to leave an FRT `scar` [35], [37]. Bacteriophage P22 HT105 int- was used as described previously [38]. The *flhB* fusion chimera genes were derived from *A. aeolicus* or *B*. *subtilis* genomic DNA and *S. typhimurium* genomic DNA by overlap extension PCR [39]. The *flhB* fusion genes were inserted into plasmid pTrc99A-FF4 [40]. Plasmid pMM26 (pTrc99A-FF4 carrying the *S. typhimurium flhB* gene) has been described previously [41]. Site-directed mutagenesis was performed using a Quikchange Lightning Site-Directed Mutagenesis Kit (Agilent Technologies), and plasmid pMM130 (pTrc99A-FF4 carrying the *S. typhimurium flhA* gene) was used as template [21]. Strain JR501 is a restriction-deficient *Salmonella* strain, which was used to modify plasmids for *Salmonella* compatibility [42].

### Affinity Western Blotting

For detection of FlhB proteins from whole cell lysates, fresh colonies of *S. typhimurium* containing the desired plasmid were inoculated into 6 ml LB with antibiotic and grown with shaking at 37°C until an OD_600_ 0.8 and induced for 2 hours with the addition of 1 mM isopropyl β-D-1-thiogalactopyranoside (IPTG). Cells were harvested by centrifugation at 16,100×*g* for 5 minutes, and the pellets were re-suspended in SDS-PAGE loading buffer (100 mM Tris-HCl [pH 6.8], 2% sodium dodecyl sulfate [SDS], 1 mM β-mercaptoethanol, 7 M urea, and 0.1% bromophenol blue) and incubated at 95°C for 15 minutes. An equivalent amount of each whole cell lysate was separated by SDS PAGE and proteins were transferred to polyvinylidene fluoride (PVDF) membranes. The polyclonal antibodies were produced in rabbits inoculated with purified *S*. *typhimurium* FlhB protein or with purified *A. aeolicus* FlhB protein. Serum was used in immunoblotting at a 1∶20,000 dilution. Primary antibodies were detected using the anti-rabbit WesternBreeze Chromogenic Kit (Invitrogen), according to manufacturer`s instructions.

### Swarm Plates, and Isolation of Motile Suppressor Mutants

Soft tryptone motility agar (0.35% [w/v]) was used in motility assays at 30°C [43]. To isolate motile suppressor mutants, 10 µl of an overnight culture was inoculated as a streak into soft tryptone agar, and after incubation suppressor mutants were purified from motility halos. Isopropyl β-D-1-thiogalactopyranoside (IPTG) was not used to induce proteins from pTrc99A-based plasmids.

### Whole Genome Sequencing

An Illumina Genome Analyzer IIx (San Diego, USA) instrument was used for parallel sequencing of the 4.9 Mbp whole genome of the parent control strain MKM50 and 6 motile suppressor mutants. A multiplexed 76-bp single-end read genomic DNA sequencing run was used. 530-Mbp data was obtained for each genome. The library samples were prepared according to Illumina instructions for multiplexing. Firstly, cellular DNA was extracted from 1.5×10^9^ bacterial cells for each sample using the SIGMA GenElute Bacterial Genomic DNA Kit. The DNA was fragmented using a Covaris S2 (acoustic sonicator) into short (about 300-bp) fragments. Other consumables used in the library preparation and sequencing run were from Illumina and used according to manufacturer`s instructions.

### Data Analysis of Whole Genome DNA Sequencing

Data processing of the sequence output from the Illumina Genome Analyzer IIx was first performed using the Illumina Genome Analyzer Pipeline software. This incorporated Sequencing Control Software Real Time Analysis (SCS/RTA) for image analysis and base calling. This was followed by Consensus Assessment of Sequence and Variation (CASAVA) software for demultiplexing, and Efficient Large-Scale Alignment of Nucleotide Databases (ELANDv2) (mismatch = 1) for further base calling. Genome Analyzer Pipeline sequence data was then analyzed using Mapping and Assembly with Quality (MAQ) and Integrative Next-Generation Genome Analysis Pipeline (inGAP) sequence analysis software to identify single nucleotide polymorphisms, insertions, and deletions for the sequenced genomes compared to the *S. typhimurium* str. LT2 genome reference sequence (NC_003197.1; Typhimurium.fasta) [44], [45].

### Microscopy of Cells

*Salmonella* cells were prepared from cultures grown at 37°C in 5 to 10 ml LB to an OD_600_ 0.6. For transmission electron microscopy, the cells were re-suspended in 10 mM MgCl_2_ and then immobilized on Mextaform HF-34 200-mesh carbon-coated copper grids. They were then stained with 4% uranyl acetate and visualized using a Jeol JEM-1230R transmission electron microscope at 100 KeV. For phase contrast microscopy, an Olympus BX53 system microscope (Japan) was used with a DP72 digital camera at 60× magnification.

### Measurement of the Quinone Pool

The cellular quinone pool was characterized using a method similar to previously described [46]. Briefly, cultures were grown aerobically at 37°C in 10 ml or 40 ml LB to an OD_600_ 0.6 for exponential phase or to an OD_600_ 3.0 to 4.0 after 24 hours for stationary phase. Culture broth (4 ml) was added to 12 ml ice-cold methanol containing trifluoroacetic acid (0.1% [v/v]). Hexane (12 ml) was added to the methanol-water mixture and vortexed for 1 minute. After centrifugation of samples at 900×*g* for 2 minutes the upper hexane phase was transferred to a clean polypropylene tube. Another 12 ml hexane was added to the methanol-water layer, and the extraction process was repeated. Hexane extracts were combined and dried in a vacuum concentrator. The dried samples were dissolved in 50 µl ethanol/dichloromethane 90∶10 and centrifuged (13400×*g*, 5 minutes). The clean samples were immediately analyzed by reversed-phase high-performance liquid chromatography (reversed-phase HPLC) (Paradigm MS4, Michrom Bioresources, Inc.). An ODS (150×2.1 mm, 5 µm, Hypersil Gold) column was used using acetonitrile/ethyl acetate (90∶10) as solvent (isocratic elution). A 10 µl loop was used and 5 µl sample was injected using an auto-sampler (CTC PAL). The flow rate used was 300 µl/min and eluted quinones were detected by a UV-detector at 276 nm. The amount of quinone (ubiquinone-8, 2-octaprenyl-6-methoxy-1,4-benzoquinone, or menaquinone-8) was calculated from the area below a chromatographic peak using a standard calibration curve of ubiquinone-10.

The chromatographic peaks were characterized by nano liquid chromatography-mass spectrometry (Nano LC-MS) using an LTQ Orbitrap (Thermo Scientific) equipped with the above HPLC system. An ODS (50×1 mm, 3 µm, Discovery Bio Wide Pore, Supelco) column was used using acetonitrile/ethyl acetate (90∶10) as solvent (isocratic elution). The flow rate used was 50 µl/min. A 5 µl loop was used and 2 µl sample was injected using the auto-sampler. A splitter was set to 1–2% of 50 µl/min.

### Measurement of the Whole Cell Protein Concentration

Pellets of bacterial cells from 200 µl to 1 ml culture were obtained by centrifugation at 5000×*g* for 10 minutes, washed, and frozen. After thawing, cells were lyzed using B-PER Bacterial Protein Extraction Reagent according to manufacturer`s instructions (Thermo Scientific, USA). Complete, EDTA-free Protease Inhibitor Cocktail (Roche Applied Science, Germany) was added to the lyzate, and the lyzate was centrifuged at 16100×*g* for 5 minutes to remove cell debris. Total protein of the supernatant was quantified using the Pierce BCA Protein Assay Kit (Thermo Scientific, USA) according to manufacturer`s instructions.

### Bioinformatics

Proteins were aligned using ClustalW2 [47]. Positions of trans-membrane α-helices were predicted using Phobius and PolyPhobius [48], [49]. Protein secondary structure was predicted using Jpred 3 [50].

## Supporting Information

Figure S1 **Sequence alignment of FlhB proteins from** ***S. typhimurium*** **,** ***A. aeolicus*** **and** ***B. subtilis*** **.** Sal  =  *S. typhimurium* (National Center for Biotechnology Information [NCBI] Reference Sequence NP_460871). Aqu  =  *A. aeolicus* (NCBI Reference Sequence NP_214382). Bac  =  *B. subtilis* (NCBI Reference Sequence NP_389520). Fully conserved residues are underlined. The level of identity between the proteins are: SalFlhB/AquFlhB, 32%; SalFlhB/BacFlhB, 36%; and AquFlhB/BacFlhB 37%. The positions of four predicted trans-membrane α-helices for the FlhB proteins are shown with a yellow background. Small residues (AVFPMILW) are in red; acidic residues (DE) are in blue; basic residues (RK) are in magenta; and residues with hydroxyl- or sulfhydryl- or amine-groups (and glycine) (STYHCNGQ) are in green. The fusion sites for the AquSalFlhB or BacSalFlhB chimeras produced in this study are highlighted with a blue background.
(PDF)

Figure S2 **Expression and complementation analysis of a BacSalFlhB chimera.** (A) Cartoon representing the FlhB proteins expressed from plasmids. Sal, *S. typhimurium*, and Bac, *B. subtilis.* (B) Immunoblotting of FlhB proteins. The FlhB proteins were expressed in a Δ*flhB* null strain. FlhB naturally undergoes autocleavage into two fragments: The trans-membrane region and the N-terminus of the cytoplasmic domain (FlhB_TM + CN_); and the C-terminal region of the cytoplasmic domain (FlhB_CC_). The FlhB_TM + CN_ fragment and uncleaved FlhB of BacSalFlhB migrated faster than corresponding fragments of the SalFlhB as indicated by the two bars adjacent to the labels. An unspecified BacSalFlhB fragment migrated at about 30 kDa. BacFlhB was not detected. An asterisk indicates a non-specific band also found for cells containing empty plasmid vector. (C) The Δ*flhB* strain expressing BacSalFlhB from plasmid makes rare flagella. A representative image from a pool of 30 cells is shown.
(TIF)

Figure S3 **Sequence alignment of FlhA proteins from** ***S. typhimurium*** **,** ***A. aeolicus*** **, and** ***B. subtilis*** **.** Sal  =  *S. typhimurium* (National Center for Biotechnology Information [NCBI] Reference Sequence NP_460870). Aqu  =  *A. aeolicus* (NCBI Reference Sequence NP_213829). Bac  =  *B. subtilis* (NCBI Reference Sequence ZP_03591364). Fully conserved residues are underlined. The identity between the proteins are: SalFlhA/AquFlhA, 42%; SalFlhA/BacFlhA, 42%; and AquFlhA/BacFlhA, 44%. The positions of eight predicted trans-membrane α-helices for the FlhA proteins are shown with a yellow background. Small residues (AVFPMILW) are in red; acidic residues (DE) are in blue; basic residues (RK) are in magenta; and residues with hydroxyl- or sulfhydryl- or amine-groups (and glycine) (STYHCNGQ) are in green. The residues aligned with the suppressor mutations in *S*. *typhimurium* FlhA found in this study, at positions Ala106 and Leu245, are highlighted with a blue background.
(PDF)

Figure S4 **Secondary structure prediction of 4-hydroxybenzoate octaprenyltransferase from** ***S. typhimurium*** **.** National Center for Biotechnology Information [NCBI] Reference Sequence NP_463099 was used. The positions of seven predicted trans-membrane α-helices are shown with a yellow background. The pink “H” represents predicted α-helical structure, and the orange “E” is predicted extended β-sheet structure. The glutamine residue at position 253, which was changed to proline through a spontaneous suppressor mutation in this study, is highlighted with a turquoise background.
(PDF)

Table S1 **Oligonucleotides used in the strain and plasmid constructions.**
(DOC)
