# Supplementary material for: Cross-Complementation Study of the Flagellar Type III Export Apparatus Membrane Protein FlhB
Source: PLoS One. 2012 Aug 29;7(8):e44030. doi: 10.1371/journal.pone.0044030 (PMC3430611; doi:10.1371/journal.pone.0044030)
Supplement: Figure S1 — Sequence alignment of FlhB proteins from S. typhimurium , A. aeolicus and B. subtilis . Sal = S. typhimurium (National Center for Biotechnology Information [NCBI] Reference Sequence NP_460871). Aqu = A. aeolicus (NCBI Reference Sequence NP_214382). Bac = B. subtilis (NCBI Reference Sequence NP_389520). Fully conserved residues are underlined. The level of identity between the proteins are: SalFlhB/AquFlhB, 32%; SalFlhB/BacFlhB, 36%; and AquFlhB/BacFlhB 37%. The positions of four predicted trans-membrane α-helices for the FlhB proteins are shown with a yellow background. Small residues (AVFPMILW) are in red; acidic residues (DE) are in blue; basic residues (RK) are in magenta; and residues with hydroxyl- or sulfhydryl- or amine-groups (and glycine) (STYHCNGQ) are in green. The fusion sites for the AquSalFlhB or BacSalFlhB chimeras produced in this study are highlighted with a blue background. (PDF) [file pone.0044030.s001.pdf]

```
                                          Transmembrane α-helix 1
Sal   -----MAEESDDDKTEAPTPHRLEKAREEGQIPRSRELTSLLILLVGVCIIWFGGESLAR 55
Aqu   --------MAEEHKTERATPYKRRKVREEGNVAKSHEIASSLVVLLSLLLLLFLGTYIAK 52
Bac   MKLRVDLQFFAGEKTEKATPKKRKDTRKKGQVAKSSDVNTAVSLLVIFLSLIAIGPYMRD 60

                                          Transmembrane α-helix 2
Sal   QLAGMLSAGLHFDHRMVNDPNLILGQIILLIKAAMMALLPLIAGVVLVALISPVMLGGLI 115
Aqu   ---EVILIFLAVTGYVHADISELGSLYENFYENIVKVLTPLFFLALLVVILSHVAQFGFI 109
Bac   RLLSFIETFYTESLTMKLSESNVHTLFVSLLKDMGMILAPILLVALVAGVVSNYMQVGFL 120

                                      Transmembrane α-helix 3
Sal   FSGKSLQPKFSKLNPLPGIKRMFSAQTGAELLKAVLK-STLVGCVTGFYLWHHWPQMMRL 174
Aqu   FTLKPLSFKWERINPFEGIKRLISLTTLFETVKNTLKAFLLIGIAVFVLKGSLYFFLSSS 169
Bac   FSAEVIQPKLEKLDPIKGFKRIYSMRAIVELIKSILK-IVVVGFAAFAVLWLHYGEILRL 179

                    Transmembrane α-helix 4  Fusion site
Sal   MAESPIVAMGNALDLVGLCALLVVLGVIPMVGFDVFFQIFSHLKKLRMSRQDIRDEFKES 234
Aqu   TYPLAETLKSFIKTSAITLITLGVVALLIAF-LDYAFKRWQYEKKIMMSRRELKEEYKQL 228
Bac   PLLTPEEALSFVSKLTLWMGLSGAGALLILAGLDYLYQRFDYEKNIKMSKQDIKDEYKKS 239


Sal   EGDPHVKGKIRQMQRAAAQRRMMEDVPKADVIVTNPTHYSVALQYDENKMSAPKVVAKGA 294
Aqu   EGHPEVKSRIKARMRELAKSRMMAEVPKATVVITNPTHIAIALKYNPEKDKAPVVVAKGK 288
Bac   EGDPIIKSKIKQRQREMAMRRMMQEVPKADVIITNPTHYAIALKYDEEKMDAPYIVAKGV 299


Sal   GLIALRIREIGAEHRVPTLEAPPLARALYRHAEIGQQIPGQLYAAVAEVLAWVWQLKRWR 354
Aqu   GTIAQKIVEIAENYSIPVVRKPELARALYPAVEVGKEISPKFYKAVAEIIAYVMFKKKKV 348
Bac   DHLALKIRKIAKEHDVMMVENRPLARALYDQVEIDQAVPEEFFKVLAEILAYVYKTKQKV 359


Sal   LAGGQRPPQPENLPVPEALDFMNEKNTDG 383
Aqu   YA--------------------------- 350
Bac   Y---------------------------- 360
```
