# Supplementary figures and images for: Cross-Complementation Study of the Flagellar Type III Export Apparatus Membrane Protein FlhB
Source: PLoS One. 2012 Aug 29;7(8):e44030. doi: 10.1371/journal.pone.0044030 (PMC3430611; doi:10.1371/journal.pone.0044030)

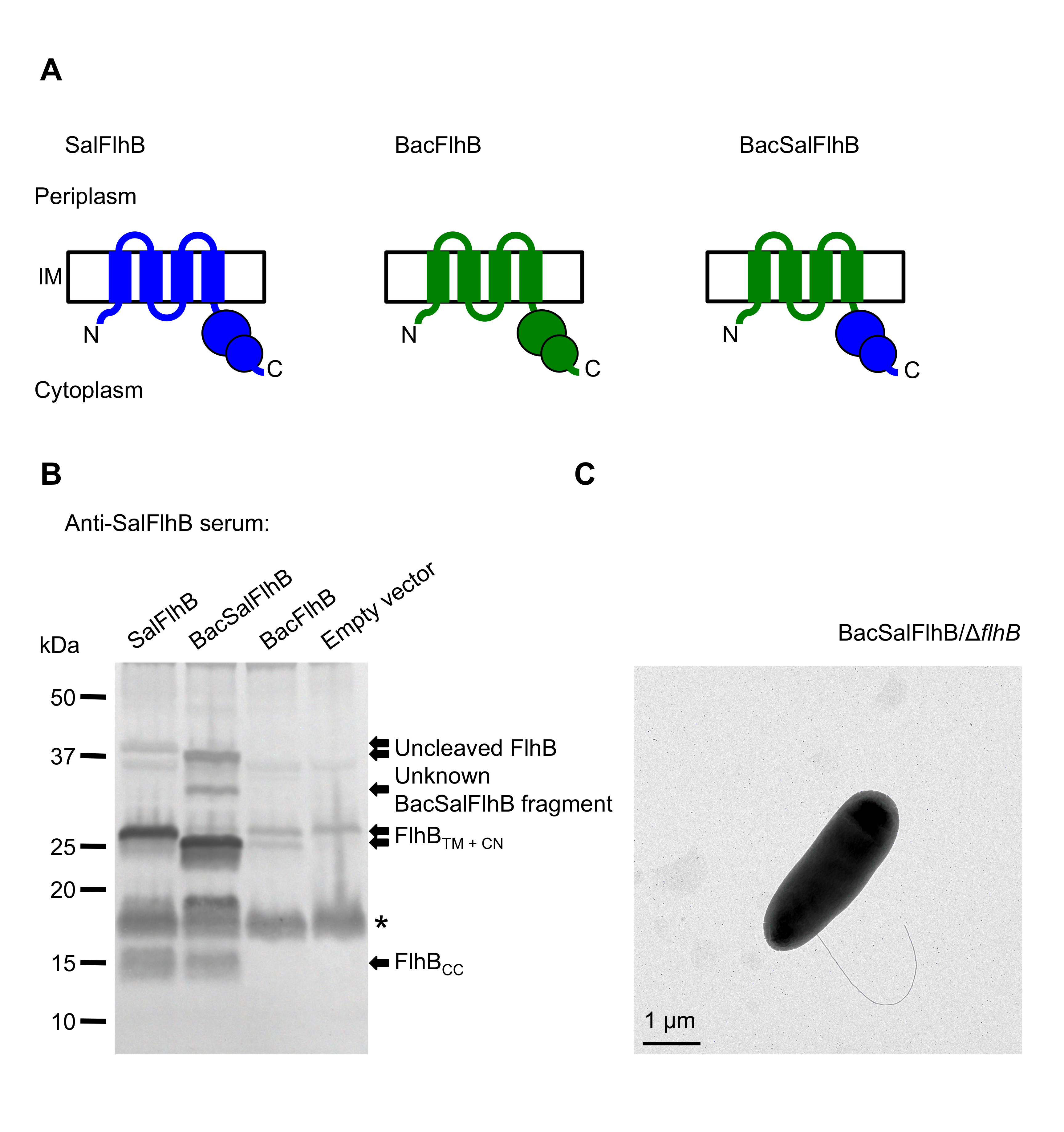

Supplement: Figure S2 — Expression and complementation analysis of a BacSalFlhB chimera. (A) Cartoon representing the FlhB proteins expressed from plasmids. Sal, S. typhimurium, and Bac, B. subtilis. (B) Immunoblotting of FlhB proteins. The FlhB proteins were expressed in a ΔflhB null strain. FlhB naturally undergoes autocleavage into two fragments: The trans-membrane region and the N-terminus of the cytoplasmic domain (FlhBTM + CN); and the C-terminal region of the cytoplasmic domain (FlhBCC). The FlhBTM + CN fragment and uncleaved FlhB of BacSalFlhB migrated faster than corresponding fragments of the SalFlhB as indicated by the two bars adjacent to the labels. An unspecified BacSalFlhB fragment migrated at about 30 kDa. BacFlhB was not detected. An asterisk indicates a non-specific band also found for cells containing empty plasmid vector. (C) The ΔflhB strain expressing BacSalFlhB from plasmid makes rare flagella. A representative image from a pool of 30 cells is shown. (TIF) [file pone.0044030.s002.tif]
