# Supplementary material for: Cross-Complementation Study of the Flagellar Type III Export Apparatus Membrane Protein FlhB
Source: PLoS One. 2012 Aug 29;7(8):e44030. doi: 10.1371/journal.pone.0044030 (PMC3430611; doi:10.1371/journal.pone.0044030)
Supplement: Figure S3 — Sequence alignment of FlhA proteins from S. typhimurium , A. aeolicus , and B. subtilis . Sal = S. typhimurium (National Center for Biotechnology Information [NCBI] Reference Sequence NP_460870). Aqu = A. aeolicus (NCBI Reference Sequence NP_213829). Bac = B. subtilis (NCBI Reference Sequence ZP_03591364). Fully conserved residues are underlined. The identity between the proteins are: SalFlhA/AquFlhA, 42%; SalFlhA/BacFlhA, 42%; and AquFlhA/BacFlhA, 44%. The positions of eight predicted trans-membrane α-helices for the FlhA proteins are shown with a yellow background. Small residues (AVFPMILW) are in red; acidic residues (DE) are in blue; basic residues (RK) are in magenta; and residues with hydroxyl- or sulfhydryl- or amine-groups (and glycine) (STYHCNGQ) are in green. The residues aligned with the suppressor mutations in S. typhimurium FlhA found in this study, at positions Ala106 and Leu245, are highlighted with a blue background. (PDF) [file pone.0044030.s003.pdf]

```
Sal    MANLVAMLRLPSNLKSTQWQILAGPILILLILSMMVLPLPAFILDLLFTFNIALSIMVLL 60
Aqu    -------------MRGKEFWIILA---VVLILSAIIIPIPALLLDLLLTLSITFSLTVLL 44
Bac    -------------MSTRDLSVLIS---VVLIVAMLVIPFPPWLLSILIIINISLALIVLL 44

Sal    VAMFTQRTLDFAAFPTILLFTTLLRLALNVASTRIILMEGHTGAAAAGKVVEAFGHFLVG 120
Aqu    LTFFIKNPLEFSSFPSVLLLGTLLRLSLNIAAARRILLHGHEGTHAAGKVIEAFGKLVAG 104
Bac    TTMNMQEALQFSIFPSLLLLLTLFRLGLNVSTTRSILSHGEG-----GKVVETFGNFVVG 99

Sal    GNFAIGIVVFIILVIINFMVITKGAGRIAEVGARFVLDGMPGKQMAIDADLNAGLIGEDE 180
Aqu    GDVVVGLIVFLIFIVINFIVITKGAERISEVAARFTLDALPGKQMSIDADLNAGLITEEE 164
Bac    GNVLVGLVVFIILIIIQFIVITKGAERVSEVAARFTLDAMPGKQMSIDADLNAGMITEQE 159

Sal    AKKRRSEVTQEADFYGSMDGASKFVRGDAIAGILIMVINVVGGLLVGVLQHGMSIGSAAE 240
Aqu    AKRRRQELEKEANFYAAMDGASKFIRGDAIAALIILFLSLVGGLLIGIGIRGMDLASAVK 224
Bac    AKHRREKVAREADFYGAMDGASKFVKGDAIAGIIIVMINIIFGIVIGMLQQGMSIQEAAS 219

Sal    SYTLLTIGDGLVAQIPALVISTAAGVIVTRVSTDQDVGEQMVGQLFSNPRVMLLAAAVLG 300
Aqu    TYTILSIGEGLASQVPALLLSTAAGVLTTKMSSRENLGEAISEELTKQPRALLFSAGVLG 284
Bac    HFTMLTVGDGIVSQIPALLISTATGIVVTRAASEGNLGHDITGQLFAYPKLLYVAAATIM 279

Sal    LLGMVPGMPNLVFLLFTAALLGLAWWLR--GREEKAPEEPQPVKMPENNSVVEATWNDVQ 358
Aqu    FIGLIPGLPTLPFLAMSAILAGAYYLVQQSLKERE-LKELEKLAQEKTKEKEEEGEELIP 343
Bac    LLGIFTPIGILLTGPLAGLLAFGAYTLSKSGKEKEEVDEILEEEAEVDELKSPESVVQLL 339

Sal    LEDSLGMEVGYRLIPMVDFQQDGELLGRIRSIRKKFAQDMGFLPPVVHIRDNMDLQPARY 418
Aqu    QPEPITLEIGYALIPLVDESQGGQIPKKIKNLRKQIAKEYGVIVPLIHIRDNLRLKPNEY 403
Bac    HIDPIEFEFGYGLIPLADANQGGDLLDRIVMIRRQLALELGLVIPVVRIRDNIALQPNEY 399

Sal    RILMKGVEIGSGDAYPGRWLAINPGTAAGTLPGEKTVDPAFGLDAIWIESALKEQAQIQG 478
Aqu    RILIKGIEIDRYELMPGHYLAVNLGNAKGPIEGIETYDPAFKIKAYWITEDKKEEAQKLG 463
Bac    RLKIKGNEVAKGELLLDHYLAMSPTPEDDLIEGIETVEPSFGLPAKWISEAVKDEADMLG 459

Sal    FTVVEASTVVATHLNHLIGQFSAELFGRQEAQQLLDRVSQEMPKLTEDLVPGVVTLTTLH 538
Aqu    YMVVDAETVLITHLSEVIKRNLHELLTRNEVMELIEMLSKKYPKVVKEIVPEQVPISIIH 523
Bac    YTVVDPASVVSTHITEKIKQHAHELIGRQETKQLIDHLKESYPVLVEEVTPNPLSVGDIQ 519

Sal    KVLQNLLAEKVPIRDMRTILETLAEHAPLQSDPHELTAVVRVALGRAITQQWFPGNEEVQ 598
Aqu    RVLQNLLREGIPVNDLLTILETLADYIEQTKDPDLLTEYVRQALSKRITRMYLT-NGTLY 582
Bac    KVLAKLLKEKVSIRNLVTIFETLADYGKLTTDSDLLTEYTRQALAKQITAQFAKENEVLK 579

Sal    VIGLDTALERLLLQALQGGG-----GLEPGLADRLLAQTQEALSRQEMLGAPPVLLVNHA 653
Aqu    AIALSPKVEAKLVKFLKENREDEFIDYVLNT---LLPKIKNEIVKFAQYGAVPVLLTSGE 639
Bac    VVTCSGRVEKAIADGVQQTEHGNYLSLEPDISESIVRSVAKEAEQLSLRQETAILLCSPP 639

Sal    LRPLLSRFLRRSLPQLVVLSNLELSDNRHIRMTATIGGK 692
Aqu    VRRFVRKTIEPYLSELAVLSYNELEKQVNIKIIGIVDED 678
Bac    VRMYVKQLLERYFPDLPVLSYNELEANVEVQSIGVVDI- 677
```
