# Supplementary material for: Cross-Complementation Study of the Flagellar Type III Export Apparatus Membrane Protein FlhB
Source: PLoS One. 2012 Aug 29;7(8):e44030. doi: 10.1371/journal.pone.0044030 (PMC3430611; doi:10.1371/journal.pone.0044030)
Supplement: Figure S4 — Secondary structure prediction of 4-hydroxybenzoate octaprenyltransferase from S. typhimurium . National Center for Biotechnology Information [NCBI] Reference Sequence NP_463099 was used. The positions of seven predicted trans-membrane α-helices are shown with a yellow background. The pink “H” represents predicted α-helical structure, and the orange “E” is predicted extended β-sheet structure. The glutamine residue at position 253, which was changed to proline through a spontaneous suppressor mutation in this study, is highlighted with a turquoise background. (PDF) [file pone.0044030.s004.pdf]

```
--------HHHHHHHHHH----HHHHHHHHHHHHHHHHH------HHHHHHHHHHHHHHH
MEWSLTQSKLLAFHRLMRTDKPIGALLLLWPTLWALWVATPGMPQLWILAVFVAGVWLMR    60

HHHHHHHHHHHH----------------EE---HHHHHHHHHHHHHHHHHHHH--HHHHH
AAGCVVNDYADRKFDGHVKRTVNRPLPSGAVTEKEARNLFVVLVLLAFLLVLTLNAMTIL    120

HHHHHHHHHHHH------H-HHHHHHHHHHHHHHHHHHHH------HHHHHHHHHHHHHH
LSVAALALAWVYPFMKRYTHLPQVVLGAAFGWSIPMAFAAVSESLPLSCWLMFLANILWA    180

HHHHHHHHHHHHHHHHH----EEHH-------HHHHHHHHHHHHHHHHHHHHH----HHH
VAYDTQYAMVDRDDDIKIGIKSTAILFGRYDTLIIGILQLGVMALMALIGWLNGLGWGYY    210

HHHHHHHHHHHHHHHH-----HHHHHHHHH--HHHHHHHHHHHHHHHH--
WAVLVAGALFVYQQKLIANREREACFKAFMNNNYVGLVLFLGLAMSYWHF              290
```
