# Supplementary material for: Cross-Complementation Study of the Flagellar Type III Export Apparatus Membrane Protein FlhB
Source: PLoS One. 2012 Aug 29;7(8):e44030. doi: 10.1371/journal.pone.0044030 (PMC3430611; doi:10.1371/journal.pone.0044030)
Supplement: Table S1 — Oligonucleotides used in the strain and plasmid constructions. (DOC) [file pone.0044030.s005.doc]

**Table S1.** Oligonucleotides used in the strain and plasmid constructions

| **Primer name** | **Sequence (5` to 3`)^a^** |
| --- | --- |
| *Primers for strain contructions*: | |
| 5-*ubiA*-*tetRA* | CCTACAAAGCCAAAATTTTCAATACGTCATATCAGTTGCCCGTTAAGACCCACTTTCACATT |
| 3-*ubiA*-*tetRA* | CGAGTAAGCCAGTGCCTGAAATGCCCGATGGCGTAAGCTTATCTAAGCACTTGTCTCCTG |
| 5-*tetRA*-*ubiE* | ATGATGAACGGGTGAAGCAGCAGTTAAGTTAACCCATACGGATTAAGACCCACTTTCACATT |
| 3-*tetRA*-*ubiE* | GATTGGGCCAATTTGCCCTACTCTGCGCCAGGACTAAACTACCTAAGCACTTGTCTCCTG |
| 5-*ispG*-*tetRA* | CGGATTGACGTGCTGCAGGTTGAAAAATAAGAACGTGATGGGTTAAGACCCACTTTCACATT |
| 3-*ispG*-*tetRA* | GGAGCCATGCGGGTTCAATCATACACGGGAAGCGCGCCGCTTCTAAGCACTTGTCTCCTG |
| 5-*flhA*::km | AAGAATACTGATGGCTAATCTGGTCGCGATGCTGCGCCTGATTCCGGGGATCCGTCGACC |
| 3-*flhA*::km | ACGCATTATTTTCCTCCAATGGTCGCCGTCATGCGGATATGTGTAGGCTGGAGCTGCTTC |
| *Primers for plasmid contructions*: | |
| 5-NdeI-AquflhB-1 | GTATTCGGAAATTCCATATGGCTGAGGAGCATAAAACAG |
| 3-BamHI-AquflhB | GTCATTAAGGATCCTATTAGGCGTAAACCTTTTTCTTTTTGAAC |
| 5-NdeI-AquflhB-2 | CATATGGCTGAGGAGCATAAAACAG |
| 3-AquSalflhB | CATGCGTAATTTCTTTTCGTACTGCCAGCGTTTG |
| 5-AquSalflhB | GGCAGTACGAAAAGAAATTACGCATGTCGCGGCAGG |
| 3-BamHI-SalflhB | GGATCCTATTAGCCATCAGTATTCTTCTCGTTC |
| 5-NdeI-BacflhB | GGAAACAGCATATGAAGCTTAGAGTTGACCTGCAG |
| 3-XbaI-BacflhB | GTCGACTCTAGATCAATATACTTTTTGTTTTGTTTTATATAC |
| 3-BacSalflhB | GACATGCGTAATTTTTTCTCATAGTCAAATCTCTGA |
| 5-BacSalflhB | GATTTGACTATGAGAAAAAAAATTACGCATGTCGCGG |
| 3-XbaI-SalflhB | GTCGACTCTAGATTAGCCATCAGTATTCTTCTCGTTC |
| *Primers for site-directed mutagenesis of plasmids*: | |
| 5-A106E | CGGCGCGGCGGAGGCGGGAAAGG |
| 3-A106E | CCTTTCCCGCCTCCGCCGCGCCG |
| 5-A106V | CGGCGCGGCGGTGGCGGGAAAGG |
| 3-A106V | CCTTTCCCGCCACCGCCGCGCCG |
| 5-L245P | AAGCTACACCCTGCCGACCATTGGCGACG |
| 3-L245P | CGTCGCCAATGGTCGGCAGGGTGTAGCTT |

^a^ Restriction sites are underlined.
